# Chemotherapy for ovarian cancer: yesterday, today and tomorrow

**DOI:** 10.1038/sj.bjc.6601493

**Published:** 2003-12-17

**Authors:** S B Kaye

**Affiliations:** 1 The Royal Marsden Hospital, Downs Road, Sutton, Surrey SM2 5PT, UK

**Keywords:** combination chemotherapy, docetaxel, ovarian cancer, QoL

Despite the many advances in the diagnosis and treatment of ovarian cancer over the past four decades, this malignancy remains the second most commonly diagnosed gynaecological cancer, claiming more lives than any other tumour of the reproductive system. One of the major challenges in treating women with ovarian cancer is that diagnosis is usually made when the disease has spread beyond the ovary. Although surgery is still considered the first-line intervention, the majority of patients present too late for curative removal of the tumour to be successfully achieved. Hence, chemotherapy remains a key part of treatment for most women with ovarian cancer.

This supplement brings together the views of several expert groups, focusing on potential ways to improve the outcome of treatment with chemotherapy.

Drs McGuire and Markman provide a thorough background and summary of current standards of care. They describe the evolution of treatment from initial single alkylating agents to current first-line therapy comprising the taxane–platinum combination. They recognise that there remains much scope for improvement. For instance, the counterintuitive and controversial results of the GOG-132 and OV010 trials (both of which indicate that the taxane–platinum combination and single-agent platinum produce equivalent results) have led to a number of new trial designs. In particular, they have encouraged investigators to consider sequential regimens, obviating the possibility of any negative interaction between the two major drug classes. Results of these various trials are awaited with interest. McGuire and Markman also review a number of novel agents for the treatment of advanced ovarian cancer, and this is certainly a very productive area. It is worth pointing out that the current best treatment of patients with optimally debulked disease typically leads to a median progression-free survival of approximately 18–20 months, and an overall median survival approaching 48 months. In other words many patients can expect to survive for more than 2 years beyond first relapse. This points to the very large potential for benefit from treatment for relapsed disease. Recent results from ICON-4 (Parmar *et al*, 2003) indicate the value of combination chemotherapy over single-agent carboplatin in this context and highlights the scope for the introduction of a range of new drugs. It also provides an ideal opportunity for the use of the alternative taxane, docetaxel, which is described in detail in the other articles.

Dr Katsumata provides a comprehensive review of the current data on the use of docetaxel in ovarian cancer. Many clinicians treating breast cancer have concluded that docetaxel is a superior agent to paclitaxel, and a recently reported randomised trial in this disease supports that view (Ravdin *et al*, 2003).

In ovarian cancer, preclinical data have indicated a lack of complete crossresistance and the activity of docetaxel in paclitaxel-refractory ovarian cancer (Verschraegen *et al*, 2000) is an important observation, not least in support of the proposal that it should be considered as a logical choice whenever retreatment with a taxane is being considered. Numerous studies have delineated the activity of docetaxel in combination with platinum as well as in single-agent trials. The most extensive is the randomised trial The Scottish Randomised Trial in Ovarian Cancer that demonstrated equivalence in terms of efficacy between paclitaxel−carboplatin and docetaxel−carboplatin in over 1000 patients. A key feature, however, was the significant difference in neurotoxicity between the two schedules and this is emphasised by several authors in this supplement. Katsumata also notes the added convenience of the 1-h schedule of docetaxel, and it is important to highlight that this offers patients the option of scalp-cooling to prevent alopecia, a manoeuvre which in the majority of cases is effective and worthwhile.

Drs Guastalla and Dieras pursue this discussion on the differences in quality of life to be anticipated from paclitaxel- and docetaxel-containing treatment in ovarian cancer. They point out that while neutropenia is more pronounced with docetaxel, this does not lead to a significant compromise in patient safety. Quality of life is an important component of all clinical trials in this disease and these authors highlight the progress being made in developing reproducible tools for its assessment.

Dr Maenpaa focuses on the most recent developments in the use of docetaxel in ovarian cancer. These include novel combinations incorporating agents such as irinotecan, topotecan, gemcitabine, vinorelbine and anthracyclines into docetaxel-containing regimens. A range of promising second-line doublets has been identified. Concurrent three-drug regimens provide more of a challenge because of additive toxicity and this provides the opportunity to explore sequential regimens, which are also discussed by other authors.

Finally, Dr Vasey provides a timely reminder that the main obstacle to further progress in chemotherapy in ovarian cancer is the development of drug resistance. He correctly points out that a thorough understanding of the underlying mechanisms is still required. This will require a concerted effort to collect appropriate tissue from treated patients, so that mechanisms relevant to the clinic rather than those described in experimental cell lines can be identified. The current availability of microarray technology to conduct comprehensive gene expression analyses offers much hope for the future.

At present, however, clinicians will continue to pursue various strategies aimed at overcoming resistance, and Dr Vasey describes several of these. They include, as discussed previously, the introduction of sequential regimens, in addition to dose-intense or intraperitoneal administration of conventional drugs. Clearly, toxicity issues will be as important as the demonstration of any improvements in efficacy, and this is particularly relevant for the intraperitoneal chemotherapy trials. Novel noncytotoxic agents also represent an important element; aberrations in cell signalling can lead to the failure of cells to undergo apoptosis and to drug resistance; a range of novel drugs are capable of reversing this — at least in experimental models.

In summary, there is now real potential for progress in the treatment of ovarian cancer and this supplement provides a number of examples of how this may be achieved.
